# Thermal, Spectral and Laser Properties of Er^3+^:Yb^3+^:GdMgB_5_O_10_: A New Crystal for 1.5 μm Lasers

**DOI:** 10.3390/ma11010025

**Published:** 2017-12-25

**Authors:** Yisheng Huang, Feifei Yuan, Shijia Sun, Zhoubin Lin, Lizhen Zhang

**Affiliations:** 1 Key Laboratory of Optoelectronic Materials Chemistry and Physics, Fujian Institute of Research on the Structure of Matter, Chinese Academy of Sciences, Fuzhou 350002, China; hysh@fjirsm.ac.cn (Y.H.); yuanff@fjirsm.ac.cn (F.Y.); ssj1027@126.com (S.S.); lzb@fjirsm.ac.cn (Z.L.); 2 University of Chinese Academy of Sciences, Beijing 100039, China

**Keywords:** laser materials, erbium ion, optical spectroscopy, lasers

## Abstract

A novel laser crystal of Er^3+^:Yb^3+^:GdMgB_5_O_10_ with dimension of 26 × 16 × 12 mm^3^ was grown successfully from K_2_Mo_3_O_10_ flux by the top seeded solution growth method. The thermal diffusivity and specific heat capacity were measured to calculate the thermal conductivity of the crystal. The absorption and fluorescence properties of the crystal at room temperature were investigated in detail. The Judd-Ofelt method was used to analyze the polarized absorption spectra. The emission cross-section of the ^4^I_13/2_→^4^I_15/2_ transition was calculated by the Füchtbauer-Ladenburg formula and the relevant gain cross-sections were estimated. Continuous-wave laser output of 140 mW at 1569 nm with the slope efficiency of 17.8% was demonstrated in a plano-concave resonator. The results reveal that Er^3+^:Yb^3+^:GdMgB_5_O_10_ crystal is a promising material for 1.5 μm lasers.

## 1. Introduction

The Er^3+^ and Yb^3+^ co-doped materials have gained much interest as laser media for emitting eye-safe laser around 1.5 μm, which can be used in many important applications, such as optical communication, environmental sensing, and medical treatments [1,2,3,4]. Many types of laser crystals co-doped with Er^3+^ and Yb^3+^ ions have been investigated and have realized 1.5 μm laser operations successfully, such as Y_3_Al_5_O_12_, Y_2_SiO_5_, KY(WO_4_)_2_, YVO_4_, YCa_4_O(BO_3_)_3_ and Sr_3_Y_2_(BO_3_)_4_ [5,6,7,8,9,10,11]. Among them, borate crystals with high phonon energy (about 1400 cm^−1^), such as YCa_4_O(BO_3_)_3_ and Sr_3_Y_2_(BO_3_)_4_, have demonstrated the most efficient laser action with slope efficiency larger than 20%. The main drawback of YCa_4_O(BO_3_)_3_ and Sr_3_Y_2_(BO_3_)_4_ crystals is their lower thermal conductivities (2.2 and 1.0 WK^−1^ m^−1^, respectively) which limit the laser power due to their tendency to crack at high pumping power [12,13]. Therefore, the studies of new borate laser crystals with a large thermal conductivity and good optical properties are needed.

Recently, the Er:Yb:LaMgB_5_O_10_ (LMB) crystal with relatively large thermal conductivity (5.0 WK^−1^ m^−1^) has been realized in a continuous-wave laser at 1567 nm with maximum output power of 160 mW and a slope efficiency about 10.1% [14]. In Er-Yb codoped crystals, a large amount (~1 × 10^21^ atoms/cm^3^) of Yb^3+^ ions are needed because the Yb^3+^ ions are considered as a sensitizer to increase the absorbed pump energy and then transfer energy to the Er ions [15]. As the ionic radius of La^3+^ (1.216 Å) is much larger than those of Er^3+^ (1.004 Å) and Yb^3+^ (0.985 Å), it is not easy for such a large amount of Yb^3+^ ions to substitute for La^3+^ ions [16]. The low concentration of Yb^3+^ ions of Er:Yb:LMB crystal may be the main cause of the low energy-transfer rates and laser efficiency. The ionic radius of Gd^3+^ (1.053 Å) ion is much closer to that of Yb^3+^ ion, so the crystal GdMgB_5_O_10_ (GMB) may provide preferable condition for the Yb^3+^ ions, a large concentration of Yb^3+^ ions in GMB crystal is possible, then a more efficiency laser crystal for 1.5 μm may be acquired. Therefore, the present work is devoted to report the thermal properties, spectral properties and continuous-wave laser performance of the Er:Yb:GMB crystal.

## 2. Experiments

**Figure 1:**
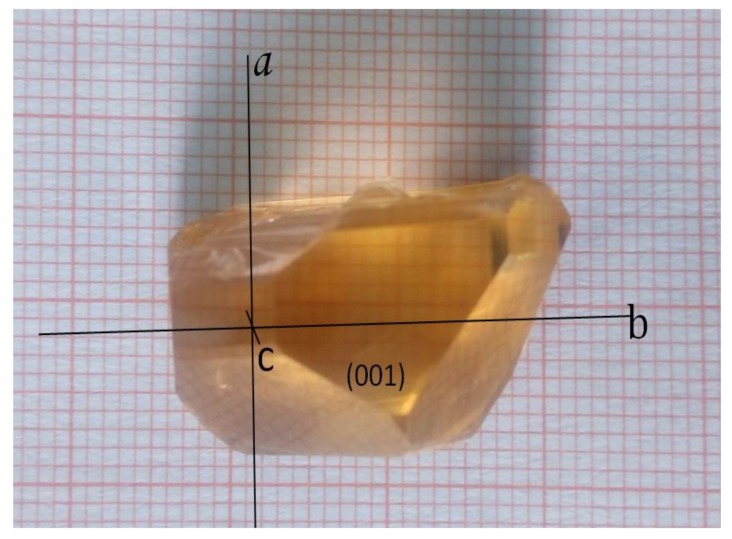
The Er:Yb:GMB crystal grown from the top seeded solution growth (TSSG) method.

The Er:Yb:GMB crystal was grown successfully by the top seeded solution growth (TSSG) method with a flux system of K_2_Mo_3_O_10_. The starting materials were weighed according to the stoichiometric composition of Er_0.03_Yb_0.20_Gd_0.77_MgB_5_O_10_ and K_2_Mo_3_O_10_ in a molar ratio of 1:2. Details of the growth procedures can be found in reference [17]. A transparent Er:Yb:GMB crystal with dimensions up to 26 × 16 × 12 mm^3^ was obtained, which is shown in Figure 1. The Er^3+^ and Yb^3+^ concentrations in the grown crystal were calculated to be 2.12 at.% (1.39 × 10^20^ atoms/cm^3^) and 12.65 at.% (8.26 × 10^20^ atoms/cm^3^), respectively, by inductively coupled plasma atomic emission spectrometry (ICP-AES, Ultima2, Jobin-Yvon). Thus, the distribution coefficients of the Er^3+^ and Yb^3+^ ions in the GMB crystal were estimated to be 0.71 and 0.63, respectively.

A square wafer of Er:Yb:GMB crystal with dimension 10.00 × 10.00 × 2.00 mm^3^ was coated with graphite on opposite sides and applied for the thermal properties measurements. The laser flash method was used to detect the specific heat and thermal diffusivity coefficient in the range of 300–550 K by using a laser flash apparatus (NETZSCH LFA457). The thermal conductivity can be calculated by the results of the specific heat and thermal diffusivity coefficient.

Since Er:Yb:GMB crystal with the monoclinic system is optically biaxial, the physical properties along the three optical indicatrix axes (X, Y, Z) are different, in which the X, Y and Z represent the three principal axes of the optical indicatrix in order of n_X_ < n_Y_ < n_Z_, respectively [17]. Two square wafers with dimensions of 5.00 × 5.00 × 2.00 mm^3^ (Z × X × Y) and 5.00 × 5.00 × 2.00 mm^3^ (X × Y × Z) were used for the measurements of the spectra. The polarized absorption spectra of Er:Yb:GMB crystal at the room temperature were measured using a Perkin-Elmer spectrophotometer (Lambda 950) in a range of 250–1650 nm. The polarized fluorescence spectra and fluorescence lifetime at the room temperature were recorded using FLS980 Fluorescence spectroscopy, which was excited by the steady state xenon lamp (Xe900, Edinburgh, UK) at 937 nm.

**Figure 2:**
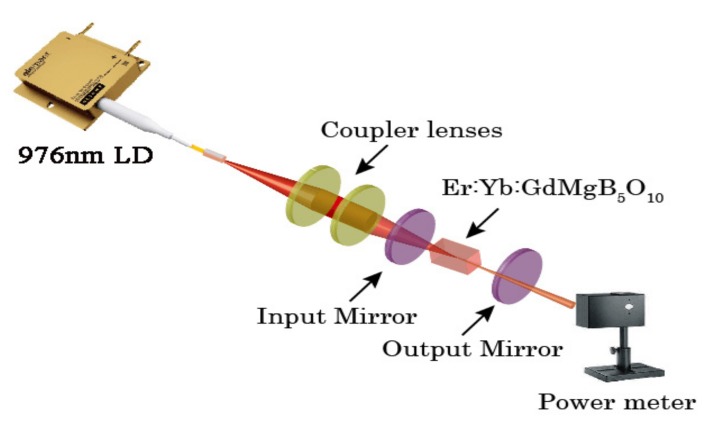
Experimental setup of LD-end-pumped Er:Yb:GMB laser.

The continuous-wave laser performance of the Er:Yb:GMB crystal was investigated in a plano-concave resonator and the experimental setup is shown in Figure 2. The sample with size of 5.00 × 5.00 × 2.00 mm^3^ was polished accurately and not anti-reflection coated. The crystal was wrapped with indium foil and then mounted in a copper holder cooled by water at 20 °C. The pumping source was a 976 nm fiber-coupled laser diode (LD) with 100 µm diameter core. The flat input mirror has 90% transmission at 976 nm and 99.8% reflectivity at 1.5–1.6 μm. Three output couplers with the different transmissions of 1.0%, 1.6% and 4.3% at 1.5–1.6 μm and identical radius curvature of 100 mm were used in the laser experiments. The resonator length was kept at about 100 mm.

## 3. Results and Discussion

### 3.1. Thermal Properties

**Figure 3:**
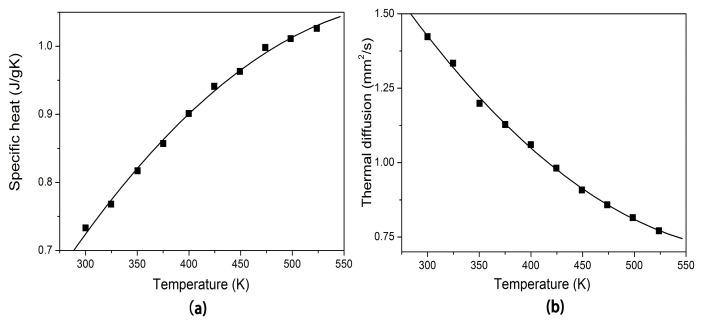
Specific heat and thermal diffusivity versus temperature curve of Er:Yb:GMB.

**Figure 4:**
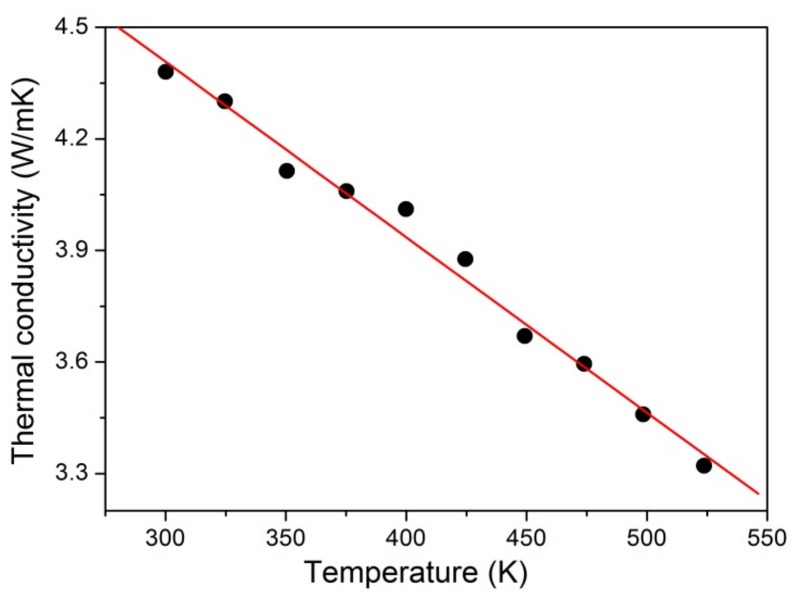
Thermal conductivity versus temperature curve of Er:Yb:GMB.

The temperature-dependence of the specific heat and thermal diffusion coefficient of Er:Yb:GMB in the temperature range 300–550 K can be found in Figure 3a,b. It can be seen that specific heat is increasing from 0.733 to 1.026 J g^−1^ K^−1^ when the temperature is increased from 300 K to 550 K. In comparison with Nd:LMB (0.655 J g^−1^ K^−1^ at 300 K) [14], YCa_4_O(BO_3_)_3_ (0.690 J g^−1^ K^−1^ at 300 K) [12] and Sr_3_Y_2_(BO_3_)_4_ (0.531 J g^−1^ K^−1^ at 300 K) [13], the Er:Yb:GMB crystal has a relatively high specific heat of 0.733 J g^−1^ K^−1^ at 300 K, so pumped laser beam can only lead to less temperature variation in the crystal and then the crystal might have high damage threshold. The thermal diffusion coefficient decreases smoothly from 1.422 to 0.771 mm^2^/s in the measured temperature range. The thermal conductivity was determined by the equation κ = λρCp, where κ, λ, ρ, Cp denote thermal conductivity, thermal diffusion coefficient, density and specific heat of the Er:Yb:GMB crystal, respectively. The ρ is 4.290 g/cm^3^ [18]. The values of thermal conductivity of the Er:Yb:GMB crystal versus temperature were calculated and shown in Figure 4. The thermal conductivity decreases from 4.380 to 3.321 W K^−1^ m^−1^ when the temperature increases. The thermal conductivity of Er:Yb:GMB is similar to that of Nd:LMB crystal as they both have an approximately linear temperature dependence, which may indicate glass-like behavior. The thermal conductivity behavior of Er:Yb:GMB is little smaller than that of (0.8 at.%) Nd:LMB crystal (5.009 W K^−1^ m^−1^ at 300 K); the reason may be the large amount of Er^3+^ ions (2.12 at.%) and Yb^3+^ ions (12.65 at.%) doped into the crystal to substitute Gd^3+^ ions. The mass substitution may increase lattice distortion to a great extent which could finally reduce the thermal conductivity of the crystal, as the thermal conductivity of YAG laser crystal declined when YAG crystal doped with a high concentration of Nd^3+^ or Yb^3+^ ions [19]. So, Er:Yb:GMB crystal has a moderate thermal conductivity (4.380 W K^−1^ m^−1^ at 300 K), which means that it is appropriate for diode-pumped continuous-wave laser operation.

### 3.2. Spectral Characteristics

**Figure 5:**
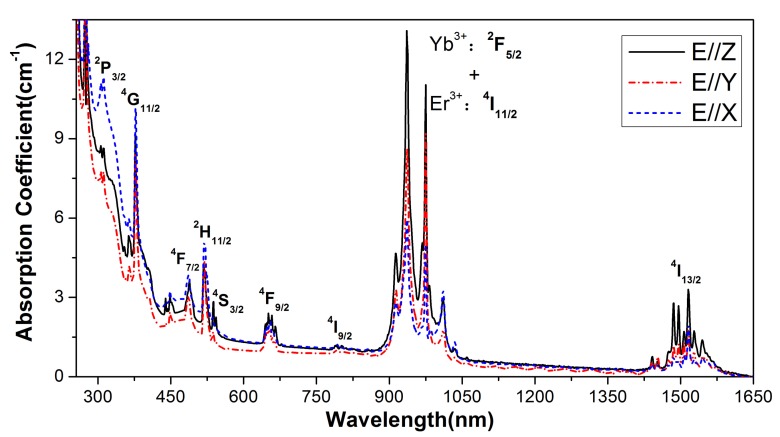
Polarized absorption spectra of Er:Yb:GMB crystal at room temperature.

Figure 5 displays the room temperature polarized absorption spectra of the Er:Yb:GMB crystal. The most intense absorption bands around 975 nm consisted by the ^2^F_7/2_→^2^F_5/2_ transition of Yb^3+^ ions and the ^4^I_15/2_→^4^I_11/2_ transition of Er^3+^ ions, and the other bands are attributed to the transitions of Er^3+^ from the ground state ^4^I_15/2_ to different excited levels which were marked in the Figure 5. The absorption cross-sections σ_abs_ is determined by σ_abs_ = α/N_c_, where α is the absorption coefficient and N_c_ is the concentration of Yb^3+^ ions in the Er:Yb:GMB crystal. Then, the peak absorption cross-sections at 975 nm were calculated as 0.66 × 10^−20^, 0.99 × 10^−20^ and 1.33 × 10^−20^ cm^2^ for E//X, E//Y and E//Z, respectively. Transition intensities of Er^3+^ ions in the GMB crystal were analyzed in the framework of the Judd-Ofelt phenomenological model [20,21]. Then the oscillator intensity parameters Ω_t_, transition probability A, fluorescence branching ratio β and radiative life time τ_rad_ were calculated. The detailed calculating process follows those of Reference [22,23]. The results are listed in Table 1 and Table 2.

**Figure 6:**
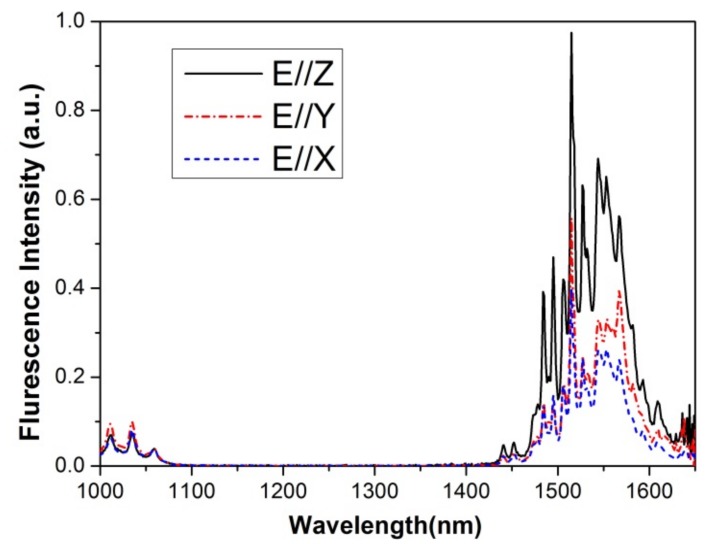
Polarized luminescence spectra of Er:Yb:GMB crystal at room temperature.

The polarized emission spectra of the ^4^I_13/2_→^4^I_15/2_ transition of the Er:Yb:GMB crystal are shown in Figure 6. The broad emission bands extend from 1435 to 1650 nm, and peak at about 1515 nm. Using the Füchtbauer-Ladenburg method, the polarized emission cross-sections σ*_em_* can be calculated by the following formula [24]: $\sigma_{em}^{F-L}(\lambda )=\lambda^{5}A_{q}I_{q}\left(\lambda \right)/\;8\pi n^{2}c\int \lambda I_{q}\left(\lambda \right)d\lambda$ where A*_q_* is transition probability of ^4^I_13/2_→^4^I_15/2_, q indicates the polarization of the fluorescence spectra, n is the refractive index of the GMB crystal, *c* is the speed of light, and *I_q_*(λ) is the relative fluorescence intensity at wavelength λ. In this work, the mean refractive index 1.40 was adopted for all the calculations. The peak emission cross-sections at 1515 nm are 0.81 × 10^−20^, 0.97 × 10^−20^ and 1.08 × 10^−20^ cm^2^ for E//X, E//Y and E//Z, respectively.

The lifetime τ_f_ of the ^4^I_13/2_→^4^I_15/2_ transition for Er^3+^ and the ^2^F_5/2_→^2^F_7/2_ transition for Yb^3+^ is about 388.7 and 10.2 μs, respectively. The efficiency of energy transfer from Yb^3+^ to Er^3+^ ions could be estimated from the formula η_ET_ = 1 − τ_f_/τ_0_, where τ_f_ is the luminescence lifetime of Yb^3+^ ions in the Er-Yb co-doped GMB crystal and τ_0_ is the lifetime in Yb-doped GMB crystal [25]. The fluorescence decay curve of a 13.08 at.% Yb:GMB crystal is about 245 μs [17]. Therefore, the efficiency of energy transfer in the Er:Yb:GMB crystal doped with 2.12 at.% Er^3+^ and 12.65 at.% Yb^3+^ is about 95.6%. Compared with η_ET_ (82%) in the (0.68 at.% Er^3+^ and 7.51 at.% Yb^3+^):LMB crystal, the η_ET_ (95.6%) in the Er:Yb:GMB crystal demonstrates higher efficiency of energy transfer from Yb^3+^ to Er^3+^ ions, which may be caused by the much larger amount of Yb^3+^ ions doped into the crystal.

**Figure 7:**
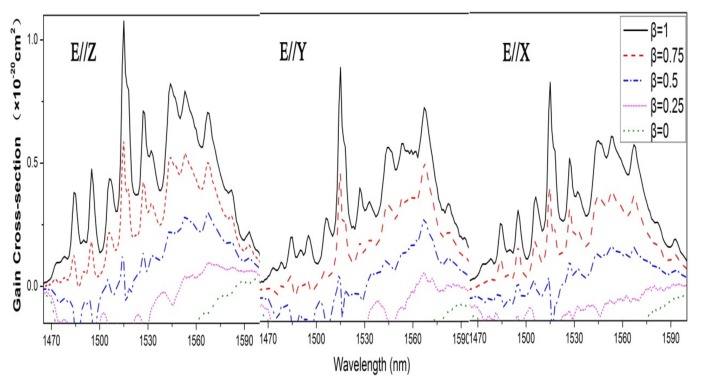
Gain spectra of the ^4^I_13/2_→^4^I_15/2_ transition of Er^3+^ ions in Er:Yb:GMB crystal for E//Z, E//Y and E//X.

The 1.5 μm laser via the ^4^I_13/2_→^4^I_15/2_ transition operates in a three-level scheme and the gain cross section σ*_gain_* can be obtained from the following equation [25]: $\sigma_{gain}(\lambda )=\beta \sigma_{em}(\lambda )-(1-\beta )\sigma_{abs}(\lambda )$ where β is the ratio of the number of Er^3+^ ions in the upper laser multiplet ^4^I_13/2_ to the total number of Er^3+^ ions. The calculated results of gain cross section with β = 0, 0.25, 0.5 0.75 and 1 are presented in Figure 7. It is inferred that for Er:Yb:GMB crystal, when the inversion parameter β is 0.5, the positive gain cross section can be achieved with the bands peak at 1567 nm, indicating that laser oscillation is possible to be realized around 1567 nm. It can also be seen that the value of σ*_gain_* for E//Z polarization is larger than those for E//Y and E//X polarizations, which indicates that the E//Z polarization is more favorable for the laser oscillation.

### 3.3. Laser Performance

**Figure 8:**
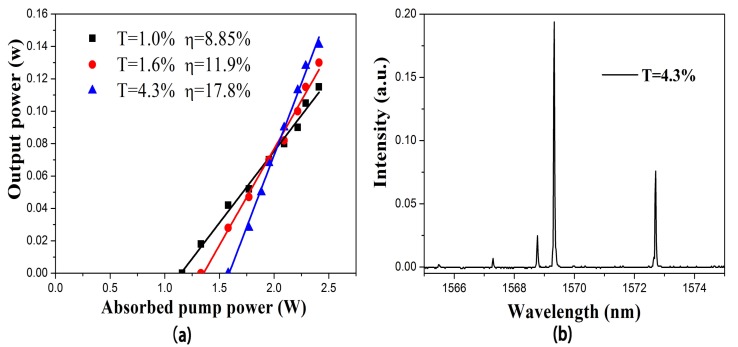
(**a**) Continuous-wave output power realized in the Er:Yb:GMB crystal for different transmissions T; (**b**) Laser spectra of Er:Yb:GMB when the absorbed pump power is 2.4 W.

An optically polished Er:Yb:GMB crystal with size of 5.00 × 5.00 × 2.00 mm^3^ (Z × X × Y), which was chosen according to the the gain cross section spectra, was used in the laser experiment. Polarization of the output laser was measured to be parallel to the Z axis. Figure 8a shows the continuous-wave output power realized in the Er:Yb:GMB crystal versus absorbed pump power for the three output couplers described in Figure 2. It can be found from Figure 8a that the best laser performance was demonstrated for the output coupler with 4.3% transmission. For this output coupler, the slope efficiency of 17.8% and output power up to 0.14 W was achieved when the absorbed power was 2.4 W. The absorbed pump threshold power, in the case, was 1.58 W. The laser spectrum measured by a monochromator (Triax 550, Jobin-Yvon) at the absorbed pump power 2.4 W and output coupler transmission 4.3% is shown in Figure 8b. The output laser spectrum with four lines around about 1569 nm was observed. At present, although the laser performance of the Er:Yb:GMB crystal is still inferior to those of Er:Yb:YCa_4_O(BO_3_)_3_ (the output power of 250 mW at 1.54 μm and slope efficiency of 21.9%) [5,11] and Er:Yb:Sr_3_Y_2_(BO_3_)_4_ (1.3 W quasi-continuous-wave output power around 1.56 μm and slope efficiency of 20%) [9,15], the slope efficiency obtained in the Er:Yb:GMB (17.8%) is larger than that of Er:Yb:GMB (10.2%) [14]. On the basis of the thermal property and initial laser experimental results, the laser slope efficiency will be further enhanced provided that the crystal optical quality is improved and laser experimental conditions are optimized. Further studies and laser experiments for Er:Yb:GMB crystal are in progress.

## 4. Conclusions

A transparent 2.12 at.% Er^3+^ and 12.65 at.% Yb^3+^ codoped GMB crystal with dimensions of 26 × 16 × 12 mm^3^ was grown successfully from K_2_Mo_3_O_10_ flux by the top seeded solution growth method. The thermal conductivity decreases from 4.380 to 3.321 WK^−1^ m^−1^ over the temperature range of 300–550 K. Its spectral properties were investigated in detail. In the framework of the Judd-Ofelt theory, the effective intensity parameters were calculated to be Ω_2_ = 5.81 × 10^−20^ cm^2^, Ω_4_ = 1.62 × 10^−20^ cm^2^ and Ω_6_ = 2.39 × 10^−20^ cm^2^. Continuous-wave laser at about 1569 nm with a maximum output power of 0.14 W and a slope efficiency of 17.8% was demonstrated. These results indicate that Er:Yb:GMB crystal is a promising laser medium for 1.5 μm lasers.

## Figures and Tables

**Table 1:** Average wavelengths, measured and calculated oscillator strengths, the intensity parameters Ω_t_ of the Er:Yb:GMB crystal.

Transition (from ^4^I_15/2_)	$\bar{\lambda }$ (nm)	E//Z		E//Y		E//X	
		f_exp_ × 10^6^	f_cal_ × 10^6^	f_exp_ × 10^6^	f_cal_ × 10^6^	f_exp_ × 10^6^	f_cal_ × 10^6^
^4^I_13/2_	1515	1.98	1.99 (ed)	2.42	2.51 (ed)	1.85	1.82 (ed)
			0.43 (md)		0.43 (md)		0.43 (md)
^4^I_9/2_	798	0.31	0.36	0.36	0.41	0.27	0.31
^4^F_9/2_	655	2.62	2.60	3.09	3.18	2.39	2.32
^4^S_3/2_	539	0.74	0.79	0.88	1.01	0.68	0.72
^2^H_11/2_	520	6.51	7.30	7.69	8.55	7.82	9.14
^4^F_7/2_	488	2.96	2.93	4.43	3.68	2.36	2.65
^4^G_11/2_	378	13.77	12.91	16.04	15.12	17.57	16.17
RMS error (%)		10.01		10.78		13.53	
Ω_t_ (10^−^^20^ cm^2^)		Ω_2_ = 4.95 Ω_4_ = 1.77 Ω_6_ = 2.26		Ω_2_ = 5.77 Ω_4_ = 2.10 Ω_6_ = 2.86		Ω_2_ = 6.69 Ω_4_ = 1.56 Ω_6_ = 2.04	
Ω_eff_ = (Ω_x_ + Ω_y_ + Ω_z_)/3, Ω_2_ = 5.81, Ω_4_ = 1.62, Ω_6_ = 2.39								

**Table 2:** Calculated radiative transition rates A, luminescence branching ratios β, and radiative lifetimes τ_r_ of the Er:Yb:GMB crystal.

J’→J	λ (nm)	E//Z		E//Y		E//X		τ_r_ (ms)
		A (s^−1^)	β (%)	A (s^−1^)	β (%)	A (s^−1^)	β (%)	
^4^I_13/2_→^4^I_15/2_	1542	151.2	100	183.8	100	141.0	100	6.30
^4^I_11/2_→^4^I_13/2_	2745	27.3	14.5	32.7	13.9	25.8	14.2	5.03
^4^I_15/2_	987	160.9	85.5	202.3	86.1	155.7	85.8	
^4^I_9/2_→^4^I_11/2_	4488	1.7	1.0	1.9	0.9	1.6	1.1	5.65
^4^I_13/2_	1703	59.7	34.2	75.8	36.0	54.2	35.0	
^4^I_15/2_	809	113.0	64.8	133.1	63.1	98.9	63.9	
^4^F_9/2_→^4^I_9/2_	3466	4.9	0.4	5.5	0.3	5.8	0.5	0.68
^4^I_11/2_	1956	83.0	5.8	103.4	6.0	79.4	6.2	
^4^I_13/2_	1142	58.8	4.1	70.8	4.1	54.9	4.3	
^4^I_15/2_	656	1137.2	89.7	1561.9	89.7	1137.2	89.1	
^4^S_3/2_→^4^F_9/2_	3125	0.9	0.04	1.1	0.04	0.8	0.04	0.46
^4^I_9/2_	1665	69.7	3.3	87.3	3.3	62.7	3.3	
^4^I_11/2_	1215	43.8	2.1	55.5	2.1	39.7	2.1	
^4^I_13/2_	842	589.2	28.1	749.3	28.2	534.8	28.1	
^4^I_15/2_	545	1390.3	66.4	1767.9	66.4	1261.9	66.5	

## References

[1] Huber G., Kranke C., Petermann K. (2010). Solid-state lasers: Status and future. J. Opt. Soc. Am. B.

[2] Krupke W.F. (2000). Ytterbium solid-state lasers. The first decade. IEEE J. Sel. Top. Quantum Electron..

[3] Tolstik N.A., Kisel V.E., Kuleshov N.V., Maltsev V.V., Leonyuk N.I. (2009). Er,Yb:YAl_3_(BO_3_)_4_-efficient 1.5 μm laser crystal. Appl. Phys. B.

[4] Bjurshagen S., Pasiskevicius V., Parreu I., Pujol M.C., Pena A., Aguilo M., Diaz F. (2008). Crystal growth, spectroscopic characterization, and eye-safe laser operation of erbium- and ytterbium-codoped KLu(WO_4_)_2_. Appl. Opt..

[5] Tolstik N.A., Troshin A.E., Kurilchik S.V., Kisel V.E., Kuleshov N.V., Matrosov V.N., Matrosova T.A., Kupchenko M.I. (2007). Spectroscopy, continuous-wave and Q-switched diode pumped laser operation of Er^3+^, Yb^3+^:YVO_4_ crystal. Appl. Phys. B.

[6] Kuleshov N.V., Lagatasky A.A., Podlipensky A.V., Mikhailov V.P., Kornienko A.A., Dunina E.B., Hartung S., Huber G. (1998). Fluorescence dynamics, excited-state absorption, and stimulated emission of Er^3+^ in KY(WO_4_)_2_. J. Opt. Soc. Am. B.

[7] Schweizer T., Jensen T., Heumann E., Huber G. (1995). Spectroscopic properties and diode pumped 1.6 μm laser performance in Yb-codoped Er:Y_3_A1_5_O_12_ and Er:Y_2_SiO_5_. Opt. Commun..

[8] Hellström J.E., Pasiskevicius V., Laurell F., Denker B., Sverchkov S., Galagan B., Ivleva L. (2005). Passive Q-switching at 1.54 μm of an Er-Yb:GdCa_4_O(BO_3_)_3 _laser with a Co^2+^:MgAl_2_O_4_ saturable absorber. Appl. Phys. B.

[9] Huang J.H., Chen Y.J., Lin Y.F., Gong X.H., Luo Z.D., Huang Y.D. (2008). High efficient 1.56 μm laser operation of Czochralski grown Er:Yb:Sr_3_Y_2_(BO_3_)_4_ crystal. Opt. Express.

[10] Gorbachenya K.N., Kisel V.E., Yasukevich A.S., Maltsev V.V., Leonyuk N.I., Kuleshov N.V. (2016). Eye-safe 1.55 μm passively Q-switched Er,Yb:GdAl_3_(BO_3_)_4_ diode-pumped laser. Opt. Lett..

[11] Wang P., Dawes J., Burns P., Piper J., Zhang H., Zhu L., Meng X. (2002). Diode-pumped cw tunable Er^3+^:Yb^3+^:YCOB laser at 1.5–1.6 μm. Opt. Mater..

[12] Luo J., Fan S., Xie H., Xiao K., Qian G., Zhang Z., Qian G., Sun R., Xu J. (2001). Thermal and Nonlinear Optical Properties of Ca_4_YO(BO_3_)_3_. Cryst. Res. Technol..

[13] Pan Z., Gong H., Yu H., Zhang H., Wang J., Boughton R. (2013). Growth, morphology and anisotropic thermal properties of Nd-doped Sr_3_Y_2_(BO_3_)_4_ crystal. J. Cryst. Growth.

[14] Huang Y., Sun S., Yuan F., Zhang L., Lin Z. (2017). Spectroscopic properties and continuous-wave laser operation of Er^3+^:Yb^3+^:LaMgB_5_O_10_ crystal. J. Alloy. Compd..

[15] Huang J.H., Chen Y.J., Lin Y.F., Gong X.H., Luo Z.D., Huang Y.D. (2013). Spectral and laser properties of Er:Yb:Sr_3_Lu_2_(BO_3_)_4_ crystal at 1.5–1.6 μm. Opt. Mater. Express.

[16] Shannon R.D. (1976). Revised effective ionic radii and systematic studies of interatomic distances in halides and chalcogenides. Acta Crystallogr..

[17] Huang Y., Lou F., Sun S., Yuan F., Zhang L., Lin Z., You Z. (2017). Spectroscopy and laser performance of Yb^3+^:GdMgB_5_O_10_ crystal. J. Lumin..

[18] Fan J., Lin Z., Zhang L., Wang G. (2007). Phase diagram, growth and spectral properties of Nd^3+^:GdMg(BO_2_)_5 _crystal. J. Alloy. Compd..

[19] Wang B., Jiang H., Zhang X., Sun H., Yin S. (2006). Study on Thermal Conductivity of the Doped GSGG Laser Crystals. J. Synth. Cryst..

[20] Judd B.R. (1962). Optical absorption intensities of rare-earth ions. Phys. Rev..

[21] Ofelt G.S. (1962). Intensities of crystal spectra of rare-earth ions. J. Chem. Phys..

[22] Li C., Wyon C., Moncorge R. (1992). Spectroscopic Properties and Fluorescence Dynamics of Er^3+^ and Yb^3+^ in Y_2_SiO_5_. IEEE J. Quantum Electron..

[23] Huang J.H., Chen Y.J., Lin Y.F., Gong X.H., Luo Z.D., Huang Y.D. (2014). Spectral properties of Er^3+^-doped CaGdAlO_4_ crystal for laser application around 1.55 μm. J. Alloy. Compd..

[24] Aull B.F., Jenssen H.P. (1982). Vibronic Interactions in Nd:YAG Resulting in Nonreciprocity of Absorption and Stimulated-Emission Cross-Sections. IEEE J. Quantum Electron..

[25] Burns P.A., Dawes J.M., Dekker P., Piper J.A., Jiang H.D., Wang J.Y. (2004). Optimization of Er,Yb:YCOB for CW laser operation. IEEE J. Quantum Electron..

